# pyBiodatafuse: extending interoperability of data using modular queries across biomedical resources

**DOI:** 10.1093/bioinformatics/btag064

**Published:** 2026-02-15

**Authors:** Yojana Gadiya, Javier Millán Acosta, Ammar Ammar, Alejandro Adriaque Lozano, Delano Wetstede, Dominik Martinát, Ana Claudia Sima, Hailiang Mei, Egon Willighagen, Tooba Abbassi-Daloii

**Affiliations:** Enveda, Boulder, Colorado, 80301, United States; Department of Translational Genomics, NUTRIM Institute of Nutrition and Translational Research in Metabolism, Maastricht University, Maastricht, 6229, The Netherlands; Department of Translational Genomics, NUTRIM Institute of Nutrition and Translational Research in Metabolism, Maastricht University, Maastricht, 6229, The Netherlands; Maastricht Centre for Systems Biology (MaCSBio), Maastricht University, Maastricht, 6229, The Netherlands; Faculty of Life Science & Technology, Leiden University of Applied Sciences, Leiden, 2333, The Netherlands; Department of Physical Chemistry, Palacky University, Olomouc, 779 00, Czech Republic; SIB Swiss Institute of Bioinformatics, Lausanne, 1015, Switzerland; Sequencing Analysis Support Core, Leiden University Medical Center (LUMC), Leiden, 2333, The Netherlands; Department of Translational Genomics, NUTRIM Institute of Nutrition and Translational Research in Metabolism, Maastricht University, Maastricht, 6229, The Netherlands; Department of Translational Genomics, NUTRIM Institute of Nutrition and Translational Research in Metabolism, Maastricht University, Maastricht, 6229, The Netherlands; Department of Medical Biology, University of Amsterdam, Amsterdam UMC, Amsterdam, North Holland, 1105, The Netherlands

## Abstract

**Motivation:**

Integrating omics data analysis with publicly available databases is crucial for unravelling complex biological mechanisms. However, this integration process is often intricate and time-consuming due to the diversity and complexity of the data involved. Achieving consistent harmonization across data types is challenging when managing disparate formats and sources. To address these issues, we introduce *pyBiodatafuse*, a query-based Python tool designed to integrate biomedical databases. This tool establishes a modular framework that simplifies data wrangling, enabling the creation of context-specific knowledge graphs (KGs) while supporting graph-based analyses.

**Results:**

We developed a pipeline for generating context-specific knowledge graphs dynamically, allowing users to create KGs on the fly from a set of gene or metabolite identifiers. *pyBiodatafuse* features a user-friendly interface that streamlines this process, making it accessible even to researchers without extensive computational expertise. Additionally, the tool offers plugins for widely used platforms such as Cytoscape, Neo4j, and GraphDB, enabling local hosting of resulting property and RDF graphs. This versatility ensures that generated KGs can be efficiently utilized within diverse research workflows. To demonstrate its potential, we used *pyBiodatafuse* to create a graph for post-COVID syndrome using differential gene expression data, showcasing its ability to build adaptable and context-specific knowledge representations. Thus, *pyBiodatafuse* sets the stage for streamlined data integration, empowering researchers to focus on discovery and analysis without being hindered by data management complexities.

**Availability and implementation:**

*pyBiodatafuse* is open-source, with its source code and PyPi package available at https://github.com/BioDataFuse/pyBiodatafuse and https://pypi.org/project/pyBiodatafuse/. The user interface can be accessed at https://biodatafuse.org/. Additionally, a release has been made on Zenodo at https://doi.org/10.5281/zenodo.18468942.

## 1 Introduction

There has been a growing interest in the generation and utility of biomedical knowledge graphs (KG), driven by the remarkable increase in available data across diverse biomedical resources (Mazein *et al.* 2024). This surge has led to the development of numerous tools and databases dedicated to constructing and refining KGs within the biomedical domain (Belleau *et al*. 2008, Chen *et al*. 2010, Bauer 2011, Williams *et al*. 2012). However, the landscape of these tools and databases is somewhat fragmented, with crucial information often spread across multiple disconnected sources. The issue of data fragmentation is further intensified by the presence of data silos, which arise due to the diverse but specific research focuses on groups across different regions of the world. These silos indirectly either limit the selection of specific tools and databases for answering questions or lead to the generation of isolated datasets or databases. This separation, in turn, limits the data accessibility and applicability, thus hindering collaborative efforts for the development of comprehensive solutions. Addressing this challenge requires efforts toward standardization, interoperability, and the breaking down of these silos to foster more effective global collaboration.

In biomedical research, data are distinguished into two types based on their source: experimental and literature-based aggregated data. Experimental data originates directly from laboratory experiments, clinical trials, or other empirical studies, yielding raw data specific to a particular context or condition. On the other hand, literature-based aggregated data is derived from published scientific literature, where researchers curate, summarize, and annotate findings from numerous studies, providing a broader understanding of various biological conditions. Despite their complementary nature, integrating experimentally generated data with literature-based data presents challenges due to differences in data formats, experimental protocols, annotation standards, data availability, and more. Bridging this divide requires sophisticated data integration techniques and robust frameworks to harmonize and contextualize disparate datasets, enabling researchers to gain deeper insights into complex biological phenomena. Consequently, initiatives and tools have emerged to address this challenge and provide resources for the community. A well-known resource in this context is StringDB (https://string-db.org/), a protein-protein interaction database (Mering 2003).

Another early example of a tool integrating biomedical resources is BioQuery (Brundege and Dubay 2003). Equipped with a standalone Java-based interface, researchers can utilize this tool to construct systematic queries that persist across database updates. A notable feature of this application was the “Query Object” framework, enabling users to formulate queries via a graphical user interface, while the application managed the intricacies of database communication. However, a limitation of the tool is its restricted access to data resources, particularly those supported by NCBI. In contrast, consortiums like Bio2RDF (Belleau *et al*. 2008), RDF Portal (Kawashima *et al*. 2018), OpenPHACTS (Williams *et al*. 2012), OpenTargets (Ochoa *et al*. 2021), and Linked Life Data (http://linkedlifedata.com/), among others, have facilitated the comprehensive collection, organization, and generation of biomedical data for community use.

While comprehensive KGs have been instrumental in consolidating diverse biomedical data sources, their construction often necessitates extensive local data caching, demanding significant storage resources that may not be universally accessible. Among these tools, InterMine (http://intermine.org/) (Smith *et al.* 2012) has stood out as particularly successful, owing to its ability to integrate data from multiple resources (and organisms) in a data warehousing approach. Building upon this concept, TargetMine (https://targetmine.mizuguchilab.org/) was developed and tailored to assist drug discovery with a special focus on target prioritization (Chen *et al*. 2011). However, despite their effectiveness, a persistent challenge lies in keeping these local data caches updated with the latest versions of the resources.

Although there is a wealth of data available in biomedical resources and an extensive storage requirement in classical KG-based solutions, not all data are directly relevant to a specific research question. This supply and demand mismatch underscores the need to carefully select and integrate data to construct a more question-specific or context-specific KG. To achieve efficient data integration, a combination of federated and modular queries can be utilized to extract relevant data subsets. Federated queries allow accessing, querying, and integrating diverse data sources without requiring data caching. However, they can be complex and data model-dependent, making maintenance challenging over time. Modular queries help mitigate this issue by breaking down the querying process into smaller, more manageable components. Together, federated and modular queries streamline data retrieval and reduce the burdens associated with traditional data storage and maintenance methods. Several other tools and data resources have since emerged, adopting concepts and frameworks inspired by the combination of federated and modular queries (Bizon *et al*. 2019, Queralt-Rosinach *et al*. 2020, Doğan *et al*. 2021, Morris *et al*. 2023). However, despite these advancements, the focus has primarily been on constructing the most comprehensive KG, historically leading to information bias based on community interests, as highlighted by Bonner *et al*. (2022). Furthermore, little attention has been given to the downstream utility of this comprehensive graph for various research domains.

To address the limitations in generating KGs, the BioCypher (https://biocypher.org/) initiative was launched (Lobentanzer *et al*. 2023). Its main mission is to democratize KG creation, enabling individuals to construct their own graphs more easily. Driven from an ontological harmonization perspective, the tool enabled the FAIRification of resources. However, the generation of graph schemas and yaml-based configuration files for data extraction is still required in the background. To further streamline and stimulate the use of KGs in the broader research community, we launched the BioDataFuse project during the ELIXIR BioHackathon Europe 2023 (Gadiya 2023). A fundamental part of this project was the *pyBiodatafuse* tool, which aimed to achieve two primary objectives: firstly, to integrate data from various biomedical resources in real-time, establishing a modular framework for data wrangling, and secondly, to provide researchers without extensive in-house data science expertise an easy-to-use pipeline to build and visualize context-specific graphs. *pyBiodatafuse* enables researchers to customize graph construction based on strategic decisions regarding their preferred data sources, thereby allowing the opportunity to reduce bias. Through *pyBiodatafuse*, we plan to assist researchers in leveraging publicly available data and integrating it with their local data in a structural, systematic, and context-specific manner.

The tool contributes to the community by addressing key aspects of integrated data analysis:

- **Extensive data collection**: The tool can integrate diverse and valuable biomedical data sources, aiming to gather comprehensive information about specific contexts.
- **Generation of KGs**: We devise a robust mechanism for structuring data presented in numerous resources into property and RDF graphs, eliminating the need for researchers to perform this task manually.
- **Data summarization**: We leverage graphical representations of data to provide visual summaries that offer quick insights into the underlying graph, particularly useful for non-graph experts. These summaries include graph-oriented statistics, such as the number and types of nodes and edges added by querying each data source, as well as research-oriented summaries, like the number of publications and patents related to the context.
- **Use case-tailored pipeline**: We have developed an end-to-end pipeline that allows researchers to upload a list of genes, compounds, or differential gene expression analysis results and generate a context-specific KG tailored to their specific needs.

Ultimately, *pyBiodatafuse* aims to advance into a FAIR-compliant platform empowering researchers to explore, interpret, and visualize data spread across diverse biomedical resources alongside internally generated data. Moreover, it seeks to integrate graph-based algorithms, encompassing both classic and machine learning approaches, enabling researchers to make novel predictions on the contextual graph. Notably, its on-the-fly query capability distinguishes it from existing approaches, eliminating the need for any local data storage.

## 2 Materials and methods

### 2.1 Design architecture and framework

**Figure 1:**
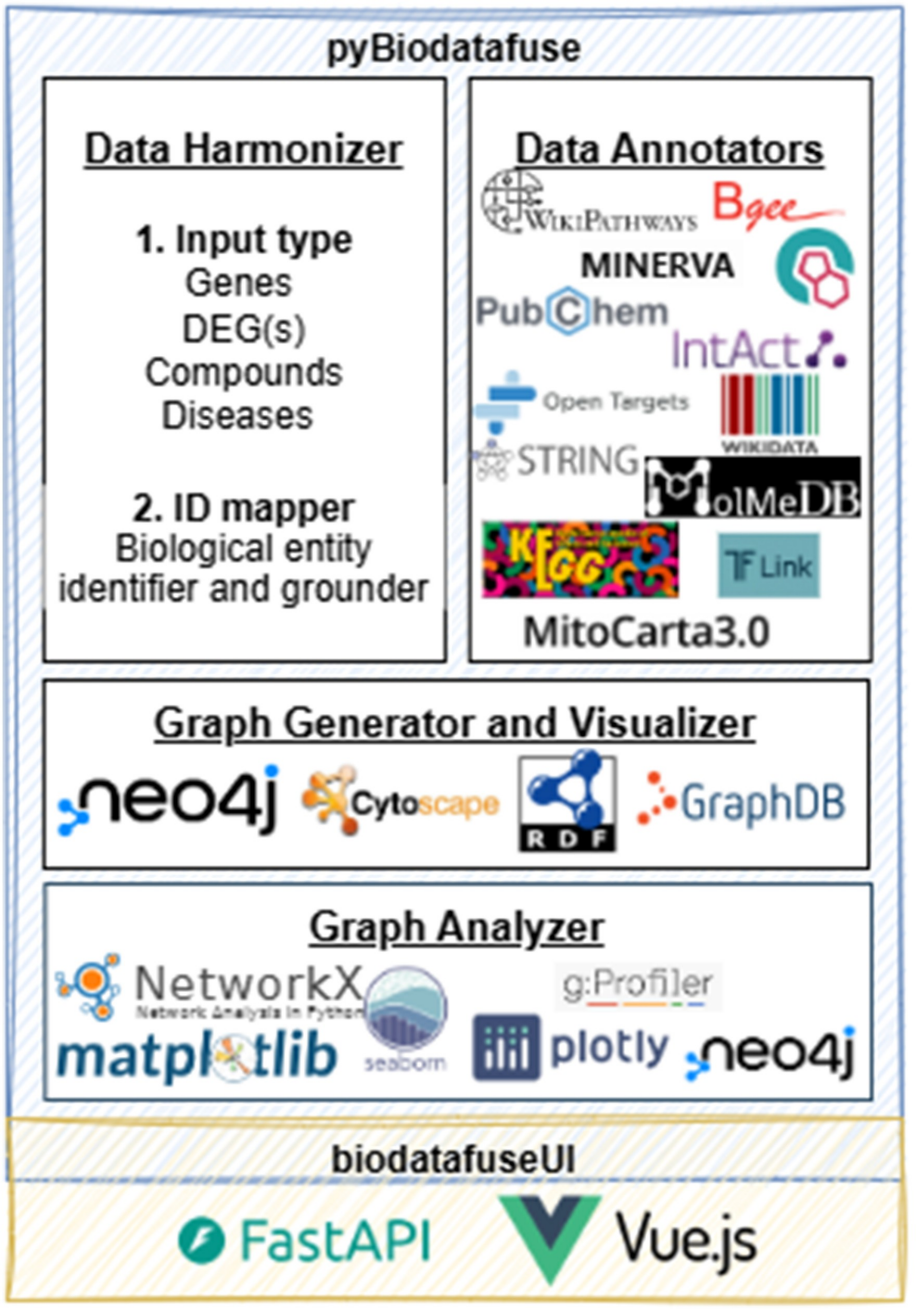
The *pyBiodatafuse* architecture and its connection with *biodatafuseUI*.

We built the *pyBiodatafuse* on four main components: (i) data harmonizer, (ii) data annotators, (iii) graph generator and visualizer, and (iv) graph analyzer (Fig. 1). In the following subsections, we elaborate on each of these components individually.

### 2.2 Data harmonizer

The data harmonizer component includes two primary elements designed to support different biological entities (genes, metabolites, or compounds) as input and an identifier mapper service. Together, these elements transform heterogeneous user-submitted data into a unified format that can be readily used by downstream “data annotators” modules. Currently, *pyBiodatafuse* supports three distinct input types: lists of genes or proteins, lists of metabolites or compounds, and the output table of a differential gene expression analysis (DEA) result table. While DEA results also contain gene identifiers, they are handled separately because they include additional metadata, such as fold changes and statistical significance, that are preserved and incorporated into the resulting KG for contextual enrichment.

Aligned with FAIR principles, our tool emphasizes the importance of unique persistent identifiers for diverse biological entities. To achieve this, the ID mapper service systematically maps biological entities from input data or the queried biomedical resources to preselected ontologies, controlled vocabularies, and terminologies. This model leverages the BridgeDb framework for database, controlled vocabulary, and ontology harmonization and mapping, enabling the *pyBioatafuse* to link identical biological concepts across different data sources (Van Iersel *et al.* 2010).

### 2.3 Data annotators

The data annotator component enables the execution of modular queries across multiple resources. The choice of resources for integration in the tool is based on their integration with ELIXIR platforms and their popularity within research communities (Drysdale *et al*. 2020). Additional factors, such as the frequency of updates and the curator community of the resource, are also considered. Rather than aggregating a large number of heterogeneous databases, *pyBiodatafuse* focuses on a curated subset of resources that are both well-maintained and broadly relevant for typical biological data analysis tasks. Currently, the tool allows users to select and query from 15 data resources (Table 1).

**Table 1:** List of biomedical data annotators in *pyBiodatafuse*.

Data resource	Query language	Supported biological entities (interaction types)
AOP WIKI RDF (Martens *et al*. 2022)	SPARQL	Genes, compounds, AOP components, GO annotations
Bgee (Bastian *et al.* 2021)	SPARQL	Genes, anatomical entities
DisGeNET (Piñero *et al.* 2020)	API	Genes, diseases
g:Profiler (Kolberg *et al.* 2023)	API	Gene, pathways
IntAct (Del Toro *et al.* 2022)	API	Genes, compounds
KEGG (Kanehisa *et al*. 2017)	API	Compounds, genes, pathways
MINERVA (Gawron *et al*. 2016)	API	Compounds, genes, pathways
MitoCarta (Rath *et al*. 2021)	–	Mitochondrial proteins, pathways
MolMeDB (Juračka *et al*. 2019)	SPARQL	Compounds, genes
OpenTargets (Koscielny *et al*. 2017)	GraphQL	Compounds, genes, pathways, biological processes, molecular functions, cellular components, diseases
PubChem (Kim *et al*. 2023)	SPARQL	Compounds, genes
STRING (Mering *et al*. 2003)	API	Genes
TFlink (Liska *et al*. 2022)	–	Transcription factors, genes
Wikidata (Vrandečić 2012, Waagmeester *et al*. 2020)	SPARQL	Genes
WikiPathways (Agrawal *et al.* 2024)	SPARQL	Genes, pathways

Each data resource utilizes its own unique storage and representation format, often requiring a specific query language for data extraction. Of these, Structured Query Language (SQL), Graph Query Language (GraphQL), Application Programming Interface (API), and SPARQL Protocol And RDF Query Language (SPARQL) are among the most widely used access mechanisms (Date 1989, Angles *et al*. 2018, Burkhardt 2020). To address this heterogeneity, *pyBiodatafuse* implements a unified querying layer that translates user queries into the appropriate format for each resource. This design enables the tool to interact with multiple resources in parallel, regardless of their underlying query language or data model. We ensure the use of consistent ontologies and terminologies for querying the resources, providing us with two main benefits: first, it enables the harmonization of biological entities within the resulting graph, thus avoiding the presence of duplicates; second, it allows us to extract data and metadata relevant to these entities, which may be distributed across multiple independent resources.

During the querying process, we store metadata about the data source and the query history itself, such as the data source version, the query size or result set size, and the query duration. This metadata provides valuable insights into the query performance and ensures reproducibility by precisely documenting the conditions and parameters of each executed query. In addition to metadata storage, *pyBiodatafuse* implements automated tests for each annotator, which are executed at regular intervals with every commit to the code base. These tests help detect breaking version updates, such as schema changes in upstream databases, ensuring that the information extracted by the tool remains accurate and up-to-date.

### 2.4 Graph generator

The graph generator component serves as the basis for generating context-enriched KGs from data captured by annotators, thus allowing for the visualization of complex connections within the dataset. The graph is primarily built using the NetworkX library (Hagberg 2020) and can be exported directly to user-desired databases and platforms such as Cytoscape (Shannon *et al*. 2003), Neo4j (Webber 2012), and GraphDB. This allows for enhanced visualization, semantic querying of complex relationships within the dataset, and interoperability with existing biomedical knowledge graphs available in RDF and SPARQL endpoints.

Our current KG supports biological entities such as genes or proteins, diseases, and metabolites or compounds. Each of these entities is represented using established ontologies and vocabularies. Entrez identifiers denote genes, UMLS identifiers identify diseases, and PubChem identifiers identify compounds. Protein-related information is currently inferred through gene identifiers and not represented as distinct entities. Furthermore, in addition to these fundamental biological entities, we incorporate location, pathways, side effects, and anatomical entities, among others, into the KG. This inclusion allows researchers to visualize subgraphs within specific contexts, such as those related to specific tissues like the heart or kidney.

### 2.5 Graph analyzer

The final component of *pyBiodatafuse* is the graph analyzer, which is designed to provide concise visual summaries of the KG. Leveraging popular Python libraries such as Matplotlib (Tosi 2009), Seaborn (Bisong 2019), and Plotly (Sievert 2020), the graph analyzer supports the creation of basic plots as well as network-specific statistical summaries. These capabilities enable users to gain a deeper understanding of the data structure and the intricate interconnections within the KG.

In addition to visualization, this module allows programmers to interact with the network using pre-defined query functions. These functions streamline the exploration and analysis process, making it easier to extract meaningful insights. The query functions include graph-centric features, including the generation of subgraphs and the preparation of training datasets for integration into existing graph algorithms. These functionalities are particularly valuable for extending the utility of the KG in machine learning and plugging it into advanced analytics workflows.

### 2.6 User interface (UI)

The complete set of functionalities offered by the *pyBiodatafuse* package is encapsulated within our web-based user interface, *BiodatafuseUI*, which is publicly accessible at https://biodatafuse.org. Designed specifically for non-technical experts, this UI enables efficient utilization of the Python package in a friendly format for non-technical users. Built using FastAPI for the backend and Vue.js for the frontend, the interface provides a user-friendly and intuitive experience for interacting with pyBiodatafuse functionalities.

### 2.7 Development guideline

To package *pyBiodatafuse*, we utilized the Cookiecutter snekpeak package (https://github.com/cthoyt/cookiecutter-snekpack), ensuring efficient and standardized packaging practices. Additionally, emphasis on documentation accompanying each function was made to enhance code reproducibility and readability. This documentation is readily accessible on Read the Docs (https://pybiodatafuse.readthedocs.io/en/latest/index.html).

To ensure streamlined functionality and detect any potential issues, we have implemented comprehensive unit tests for each annotator. These test scripts serve to maintain a stable connection between resources during querying processes. Furthermore, we have integrated Tox-based testing into our GitHub Actions, automating style, dynamic typing, and documentation tests on the codebase. This automated approach significantly streamlines the process of identifying and addressing any potential integration issues and enables community development.

### 2.8 Implementation details

*pyBiodatafuse* is developed using the Git version-control system, is written in Python, and can be found at PyPi (https://pypi.org/project/pyBiodatafuse/) and on GitHub (https://github.com/BioDataFuse/pyBiodatafuse). The current stable version of the package is 1.2.0. Additionally, the scripts associated with the UI can be assessed on GitHub (https://github.com/BioDataFuse/biodatafuseUI), and the UI is publicly accessible at https://biodatafuse.org/.

## 3 Results

The BioDataFuse project is a community-driven initiative aimed at supporting the construction of context-specific KGs and facilitating hypothesis generation based on the KG. This project involves the development of a Python package called *pyBiodatafuse* to allow for cross-talk across different biological resources and a FastAPI-based UI, *BiodatafuseUI*, to increase accessibility to domain and non-domain experts. We have crafted our data collection approach such that the generated graphs are not only rich in data sourced from various biomedical resources but also enriched with underlying metadata captured across these resources independently.

In the following sub-section, we discuss the intricacies of the data model underpinning our biomedical KG. We then illuminate the significance of the metadata-enriched KG that serves as the foundation of our primary biomedical KG and demonstrate an example case by generating the first context-specific subgraph for post-COVID-19 syndrome. Finally, we conclude the section by listing the limitations of the *pyBiodatafuse*, showcasing its potential for future growth and development in the Biomedical Knowledge Graph community.

### 3.1 Graph data model

**Figure 2:**
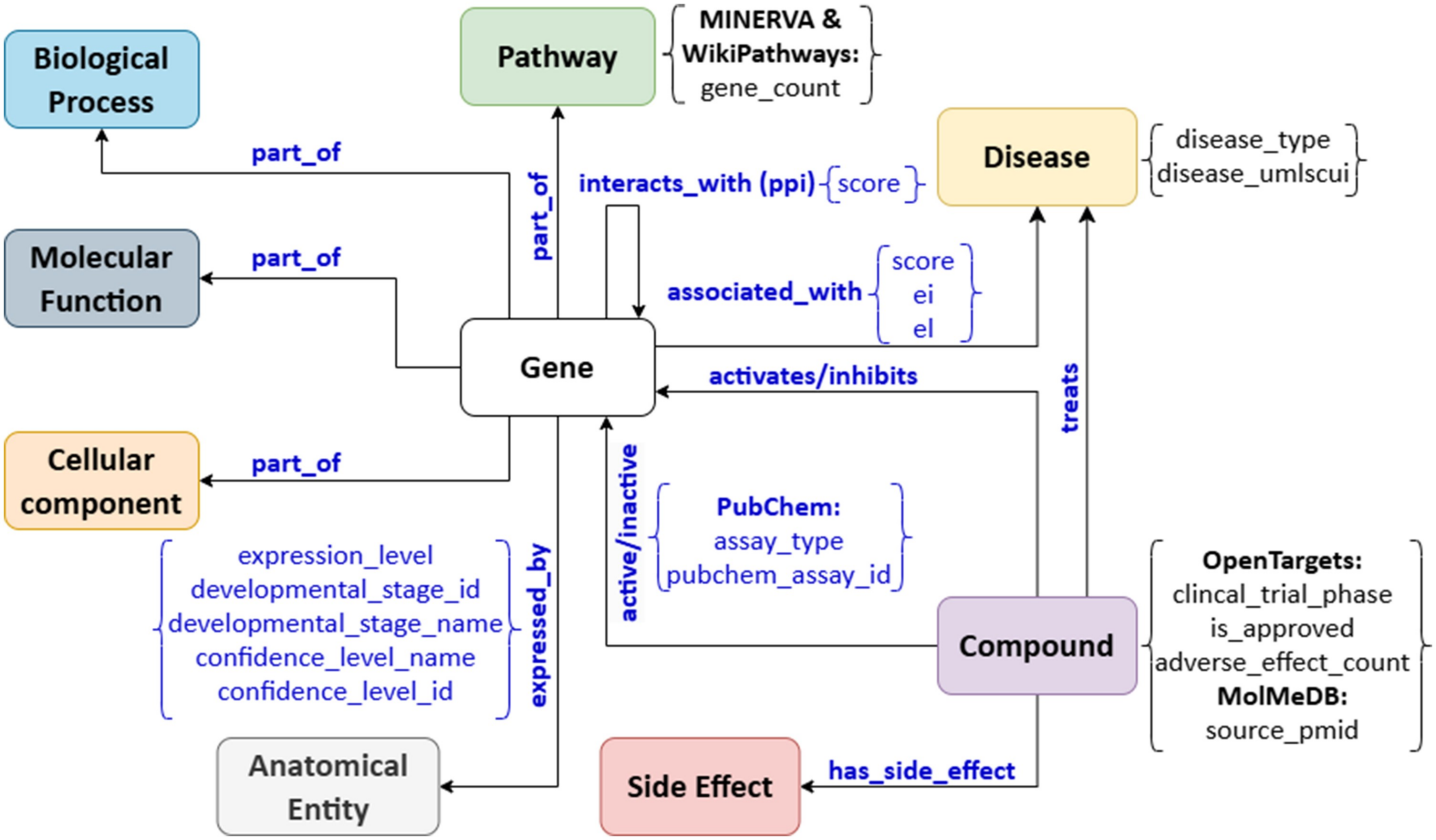
The property graph data model of the biomedical knowledge graph was generated by *pyBiodatafuse*.

We have developed a robust property graph data model connecting various biological entities in a structured manner. As shown in Fig. 2, our data model currently covers 18 distinct node types: gene, disease, compound, anatomical entity, pathway, molecular function, biological process, cellular component, side effect, homolog, phenotype, miRNA, transcription factor, mitochondrial pathway, key event, molecular initiating event, adverse outcome pathway, and adverse outcome. Furthermore, each node within the KG is enriched with attributes, also known as metadata, offering insights into its origin and context distributed across resources. For instance, disease nodes include information about the namespace and cross-references to unique identifiers in different databases and ontologies, as well as pertinent attributes like disease type. Similarly, anatomical entity nodes include details on expression levels and developmental stages, providing a comprehensive view of their biological context.

All the nodes are connected through directed edges categorized into ten types (interacts with, part of, associated with, expressed by, activates, inhibits, treats, has side effect, upstream of, and downstream of). This clear and structured representation of our KG reveals patterns, clusters, and trends embedded across the data resources. Such insights are valuable for researchers, analysts, and decision-makers, empowering them to make informed decisions. For example, the KG can provide a quick overview of the diseases, drugs, pathways, and cellular component context around a gene rather than searching for this information by accessing multiple resources. Additionally, each edge in the KG tracks provenance information, that is, the data resource it was extracted from, allowing researchers to look into the original data resource for more detailed information.

**Figure 3:**
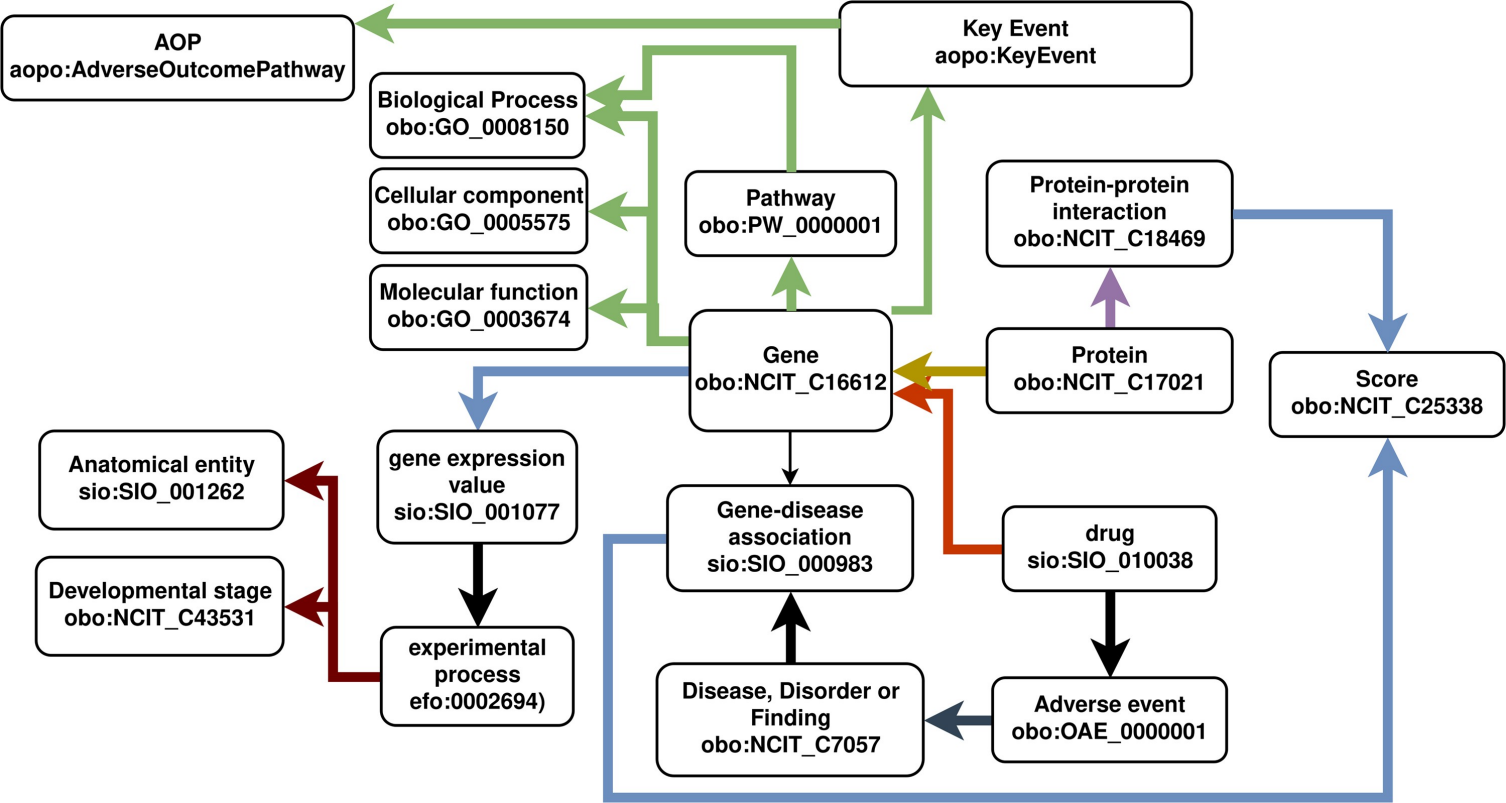
Simplified RDF schema of the biomedical knowledge graph generated through *pyBiodatafuse*. Color legend—Green: sio: has_part, blue: sio: has_measurement_value, yellow: so: translation_of, red: Relation Ontology (RO) predicates, subclasses of *Regulates activity of* (obo: RO_0011002). The detailed RDF schema for BioDataFuse RDF graphs is available as Shape Constraints Language in https://raw.githubusercontent.com/BioDataFuse/pyBiodatafuse/refs/heads/main/examples/bdf_example.shacl and as a SVG diagram under https://raw.githubusercontent.com/BioDataFuse/pyBiodatafuse/refs/heads/fixes_compoundwiki/examples/BDF_example_SHACL.svg.

Simultaneously, the graph acts as an intermediary stage, ready for serialization into three distinct formats. The first format follows Cytoscape’s specifications in JSON, enabling integration with the tool. The second format, GraphML, aligns with Neo4j’s requirements and can be loaded using Neo4j’s APOC extension. The last format is its serialization to linked data, allowing for more straightforward integration with other resources while explicitly representing the relationships and classes in the graph using established ontologies in a computer-actionable format. The linked data version of the graph (Fig. 3) has been modeled in the RDF using well-known classes and predicates from ontologies and controlled vocabularies such as the Semanticscience Integrated Ontology (Dumontier, 2014) and community staples collected in the Open Biomedical Ontologies (Jackson *et al*. 2021). We facilitate the setup of RDF triplestores with a wrapper for the GraphDB API, GraphDBManager, in order to more efficiently generate GraphDB repositories, and upload, manage, and query their RDF data. Moreover, the Shape Expressions (ShEx) or Shapes Constraint Language graph (SHACL) for the generated RDF dataset can also be automatically retrieved based on the classes used to generate the RDF version of the KG.

### 3.2 Metadata-enriched graphs

In the previous section, we explored the data model of *pyBiodatafuse* KG, which enables systematic connections between entities. While some metadata is directly incorporated into the graph data model (refer to Fig. 2), others, although not explicitly part of the schema, wield equal significance in hypothesis generation. The gene-anatomical entity edge (“expressed_in”) is peculiar in our data model, showcasing the gene expression levels (either over- or under-expressed) within specific tissues or organs. This feature contributes to a better understanding of gene functionality in a single tissue and facilitates cross-species comparisons, such as in mice. Similarly, the gene-cellular component edge (“part_of”) pinpoints cellular locations where the gene is situated, thereby helping to mitigate potential cellular toxicity effects for prospective drugs.

With the wave of open science, there is an increasing challenge for researchers to stay abreast of recent developments and publications. Even generative AI technologies like ChatGPT struggle to keep pace, occasionally resulting in hallucinations (Emsley 2023). Consequently, there is a natural inclination to revert to non-generative AI approaches to handle this. In *pyBiodatafuse*, we leverage the collaborative data curation effort in Wikidata to guide researchers toward comprehensive overviews of genes and compounds in publications. This way, the researchers can constantly monitor community interests and gauge their significance effectively.

Analogous to scientific documents, patent documents serve as crucial evidence to unravel the pharmaceutical landscape surrounding biological entities. In *pyBiodatafuse*, we harness PubChem, coupled with its integration with Google Patents, to summarize information on patents about biological entities. While both scientific publications and patents have the potential to augment the graph significantly, they have been excluded from the core data model due to their expansive nature. Nevertheless, the researchers can query these sources for selected subsets of biological entities as needed. In the case of the RDF serialization, the metadata accompanying each graph node traces back to its source, consultation or retrieval date, and API versions.

### 3.3 Context-specific graph: first long COVID KG

The COVID-19 pandemic has had devastating effects on public health, the economy, and social structures worldwide. Post-COVID syndrome (PCS), also known as Long COVID, describes a condition where individuals continue to experience a range of symptoms, including fatigue, weakness, and myalgia, even after recovering from the virus (Lopez-Leon *et al*. 2021). To better understand the indication, several graph-based databases were developed and collected into projects such as BY-COVID (https://by-covid.org/). These graphs have been prominently used by academic researchers to understand drug repositioning or repurposing for COVID. Being a niche domain in COVID-19, exploring data relevant to PCS requires manual efforts to integrate data across these resources. To reduce the manual work significantly, we employed *pyBiodatafuse* to generate a context-oriented graph for PCS.

**Figure 4:**
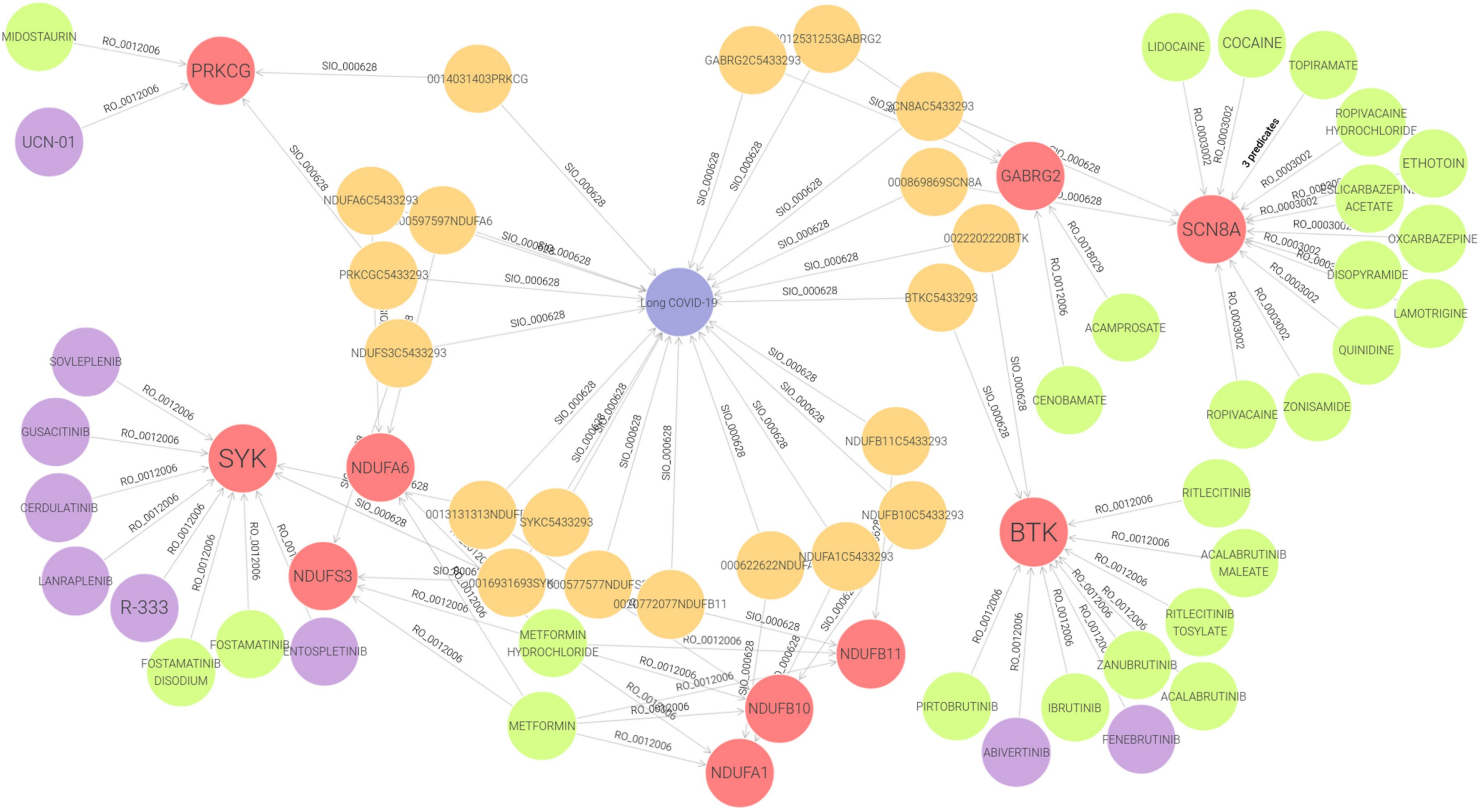
Visualization of an RDF subgraph in GraphDB. The subgraph centers on the “Long COVID” node and includes its associated gene–disease association nodes, the related genes, and drugs that inhibit, block, or act as inverse agonists of those genes. Both approved and non-approved drugs are represented. The SPARQL query used to construct the subgraph in GraphDB is available at: https://github.com/BioDataFuse/pyBiodatafuse/tree/main/examples/usecases/PCS/query_genes_drugs.rq.

We initiated the graph generation through the collection of differentially expressed genes from a transcriptomic dataset by Aschman *et al*. (2023). This dataset compared the gene expression profiles of the PCS cohort and the historical control cohort, 2023 differentially expressed genes (*P*-value < 0.05). These genes served as the starting point for the *pyBiodatafuse* tool, of which only 1667 genes were found to be consistently mapped to established ontologies. Next, we selected four data sources (DisGeNet, OpenTargets, MINERVA, and StringDB) to generate the PCS KG, resulting in five different edge types (gene-disease, gene-compound, disease-compound, gene-pathway, and gene-gene). We also included an additional disease node labelled “Long COVID-19” (UMLS:C5433293), with literature-based data aiding in connecting some input genes to the disease, reflecting insights from Das and Kumar’s study (2023). Alongside these, we incorporated gene-specific annotations from GO. The resultant graph comprises 19 345 nodes and 92 483 edges. The PCS KG has been made available in different graph formats for community use via https://github.com/BioDataFuse/pyBiodatafuse/tree/main/examples/usecases/PCS. The RDF version can be easily uploaded to any RDF graph database or endpoint. Figure 4 provides a sample visualization of part of the graph showing which drugs could potentially regulate genes involved with PCS.

### 3.4 Limitations

The *pyBiodatafuse* tool, while powerful, comes with several limitations that should be addressed for optimal use and future improvements. Firstly, the framework relies heavily on requesting data from upstream services via mechanisms such as SPARQL, APIs, GraphQL, or similar interfaces to build the KG. If an endpoint becomes temporarily unavailable, is deprecated, or is relocated, *pyBiodatafuse* cannot fetch the required data. There are currently no standardized practices for reporting new database releases, nor are such updates systematically collected by tool registries such as bio.tools. This highlights a broader community-wide gap in software dissemination and curation practices, which, while beyond the scope of this paper, warrants greater attention. This dependency can disrupt the KG generation process downstream and introduce bottlenecks; however, these incidents are caught and reported to the user in both the python package and UI versions of BioDataFuse. Secondly, each “data annotator” within the tool is highly customized to extract information from specific resources and databases. This customization necessitates a deep understanding of the underlying data models of these resources. Users without this expertise may face challenges in adapting or extending the framework to accommodate additional data sources. Thirdly, despite taking experimental data (e.g. differentially expressed genes) as input, the tool does not handle any raw data processing and expects users to preprocess their raw data presented in proprietary or non-proprietary formats. Lastly, the tool is tightly coupled with the data models of the resources it integrates. Any changes to these data models, such as schema updates, attribute renaming, or structural modifications, can significantly impact the tool’s functionality. This sensitivity requires continuous maintenance to ensure compatibility with evolving data sources. These limitations highlight the need for strategies to improve the robustness and scalability of *pyBiodatafuse*, such as incorporating redundancy in endpoint access, developing more adaptable data annotators, and implementing automated tools to detect and adapt to changes in data models.

## 4 Discussions

KGs are comprehensive representations of data and enable the capturing and harmonising of heterogeneous, multi-modal data with explicit semantics. Previously, generating such KGs required informaticians and engineering experts, thus restricting the ability to build graphs in an experimental lab setting. Frameworks like BioCypher were developed to decentralize KG generation, but these tools still require sufficient technical expertise for utility. The BioDataFuse distinguishes itself from other tools by providing a consistent modular schema, completely abstracting the knowledge engineering task from the process of generating knowledge graphs to ensure continuous integration. Since the graph is populated with the requested node types only, the resulting schema utilizes the relevant set of nodes and predicates for the customized graph.

In this work, we proposed the *pyBiodatafuse* tool that allows users to generate context-specific graphs irrespective of their technical expertise. This tool has significantly advanced the capability for on-the-fly graph building by enabling modular querying across endpoint-independent resources and databases. By eliminating dependency on specific endpoints, the tool ensures greater flexibility and adaptability in integrating diverse datasets. With its community-driven model, we aim to onboard experts and data owners to continually expand and diversify the range of data sources integrated into the tool. This collaborative approach not only enhances the breadth of data but also fosters innovation through shared expertise. In this context, we are now looking at the prospect of integration of the tool into the Biocypher community ecosystem (https://biocypher.org/).

Looking ahead, we aspire to add prediction models, such as DreamWalk (Bang *et al*. 2023), directly into our resources. This initiative will provide two key advantages: (i) wider applicability and usability of the models through Python packaging and (ii) direct plugins to model input data (i.e., graphs), allowing for completing the loop from data to predictions. Beyond prediction modeling, we plan to enrich the tool by supporting additional input types, such as outputs from gene co-expression analysis or mass spectrometry data, to broaden its analytical capabilities. Another critical enhancement will be enabling efficient cross-species data comparisons, such as between humans and model organisms like mice. This feature will significantly extend the tool’s utility across diverse biological contexts, paving the way for innovative research and discovery. An additional improvement point revealed by the addition of several different annotators, which had to extend support for a variety of controlled vocabularies, is the lack of mapping efforts between tightly connected ontologies and vocabularies, like matching the Key Events from the AOP framework to the adverse events in the Ontology of Adverse Events (He *et al.* 2014) or Gene Ontology processes. Finally, to speed up data annotator building, we envision connecting schema extraction tools such as VoID generation, RDF-Config, SheXer, and others, allowing for the generation of an annotator template in a semi-automated fashion (Millán Acosta *et al.* 2025). These planned developments position *pyBiodatafuse* as a comprehensive and versatile biological data integration and analysis framework.

## Data Availability

The data and code underlying this study are available at https://github.com/BioDataFuse/pyBiodatafuse. A permanent archived version is available at Zenodo (doi: https://doi.org/10.5281/zenodo.18468942).
